# Reference-free inference of tumor phylogenies from single-cell sequencing data

**DOI:** 10.1186/1471-2164-16-S11-S7

**Published:** 2015-11-10

**Authors:** Ayshwarya Subramanian, Russell Schwartz

**Affiliations:** 1 Department of Biostatistics, Harvard T.H. Chan School of Public Health, 655 Huntington Street, 02115 Boston, USA; 2 Department of Biological Sciences and the Computational Biology Department, Carnegie Mellon University, 5000 Forbes Avenue, 15213 Pittsburgh, USA

**Keywords:** tumor evolution, tumor phylogeny, k-mer, single-cell sequencing

## Abstract

**Background:**

Effective management and treatment of cancer continues to be complicated by the rapid evolution and resulting heterogeneity of tumors. Phylogenetic study of cell populations in single tumors provides a way to delineate intra-tumoral heterogeneity and identify robust features of evolutionary processes. The introduction of single-cell sequencing has shown great promise for advancing single-tumor phylogenetics; however, the volume and high noise in these data present challenges for inference, especially with regard to chromosome abnormalities that typically dominate tumor evolution. Here, we investigate a strategy to use such data to track differences in tumor cell genomic content during progression.

**Results:**

We propose a reference-free approach to mining single-cell genome sequence reads to allow predictive classification of tumors into heterogeneous cell types and reconstruct models of their evolution. The approach extracts k-mer counts from single-cell tumor genomic DNA sequences, and uses differences in normalized k-mer frequencies as a proxy for overall evolutionary distance between distinct cells. The approach computationally simplifies deriving phylogenetic markers, which normally relies on first aligning sequence reads to a reference genome and then processing the data to extract meaningful progression markers for constructing phylogenetic trees. The approach also provides a way to bypass some of the challenges that massive genome rearrangement typical of tumor genomes presents for reference-based methods. We illustrate the method on a publicly available breast tumor single-cell sequencing dataset.

**Conclusions:**

We have demonstrated a computational approach for learning tumor progression from single cell sequencing data using k-mer counts. k-mer features classify tumor cells by stage of progression with high accuracy. Phylogenies built from these k-mer spectrum distance matrices yield splits that are statistically significant when tested for their ability to partition cells at different stages of cancer.

## Background

Cancer remains a major public health challenge and the road to effective management of the disease is challenged by widespread inter- and intra-tumoral heterogeneity [1,2]. Tumors at the same site of origin and identical clinical presentation may show wide differences in genomic [3] and hence, functional [4] architectures, leading to a diversity of underlying cellular mechanisms, drug responses and treatment outcomes. A key insight that has informed work in this area is the recognition that a tumor is an evolutionary system [5,6], in which individual cells undergo a process of rapid mutation and selection leading to an accumulation of driver mutations and, consequently, a progression in phenotypes. Though varying rates of progression result in widespread heterogeneity, a fundamental hypothesis is that a few "driver" mutations [7] affecting key cellular pathways control any specific tumor type and that these drivers can be identified and exploited for effective and efficient disease management. Recent research [8,9] has shown that driver mutations function through clones of heterogeneous cells which progress through space and time. The search for such clonal populations defining robust progression subtypes and the paths leading to them remains an active area of research. Various experimental and in-silico approaches have been devised [10] and tumor phylogenetics (c.f., [11]) is one such strategy for interpreting the evolution of tumors using computer algorithms for phylogenetics, i.e., the inference of evolutionary trees. The result is a tumor phylogeny, or phylogenetic tree, a reconstruction of the sequences of mutations that cells within a tumor or class of tumors accumulate over the course of their progression. The trees allow us to learn markers of progression driving key steps in tumor evolution, identify and classify tumor subtypes with possibly different underlying mechanisms of action, and enable predictive modeling of future stages of progression. Much prior work has shown the utility of tumor phylogenies using data from single-cell Fluorescent In-Situ Hybridization (FISH) [12-15] and microarray technologies [16,17]. Advances in deep sequencing technology, particularly single-cell sequencing [18,19], have extended this interest by promising a granular view of heterogeneity and progression within a single tumor. Single-cell sequencing, however, results in large noisy datasets, which are computationally expensive to analyze, and extending prior methods requires customizing them at different levels to accommodate new noise models. With such massive scale data becoming rapidly available, there is a need for new strategies to exploit them to derive robust progression models.

Here, we propose a reference-free method for inferring genome evolutionary distance from single-cell sequencing data towards building tumor phylogenies. Typically, a comparison of tumor genomes would involve their alignment to the human reference genome followed by identification and calling of aberrations of interest, usually genome copy number variations (CNVs), single nucleotide polymorphisms (SNPs) or other structural variations. In the case of genome copy number changes, such a method assumes an approximation of genome copy number at the individual tumor level and makes consolidation of multi-sample copy number changes challenging. Our method does not rely on the availability of a well annotated reference genome and we draw inspiration from the widely used k-mer based reference-free methods [20-22] to derive genomic measures of copy number changes among samples. We then assign a utility to this information by using it in tasks of classification and phylogeny inference. We also demonstrate approaches for evaluating the resulting phylogenies by hypothesis-testing of splits in the tree using random null models. Besides computational simplicity, a reference-free method offers another advantage unique to the study of tumors: tumor genomes are massively rearranged and, hence, alignment to the reference genome can be a noisy process. Our method is illustrated through its application on a publicly available breast tumor single-cell sequencing dataset [18]. This dataset has 200 cells and its analysis provides a prototype for more massive data as they become available. Through this application, we conclude that our method offers advantages of (1) a stand-alone reference-free approach (2) easy comparison of multiple tumor genomes and (3) translating tumor copy number variation into practical use as features for classification and evolutionary inference.

## Methods

**Figure 1:**
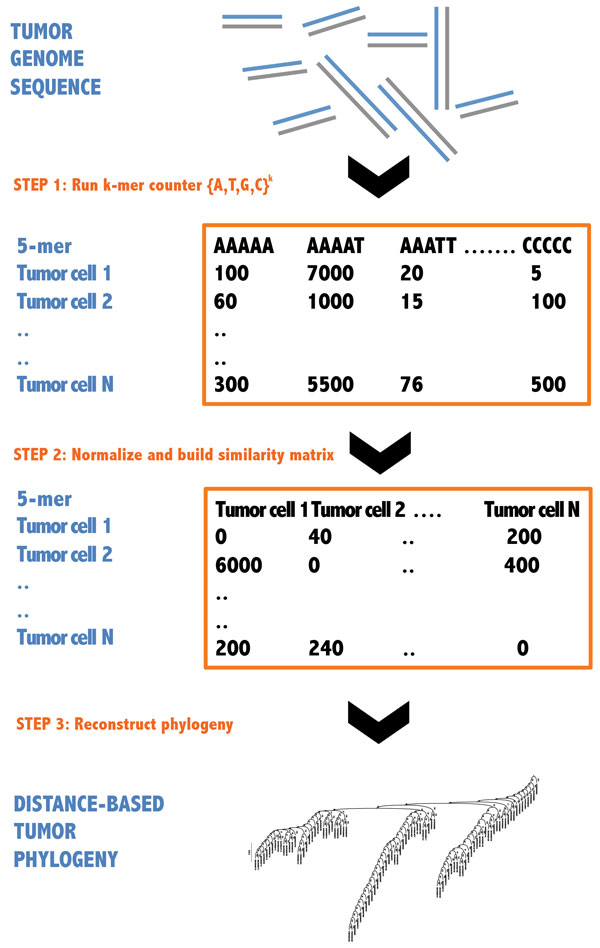
**Workflow for inferring phylogenies from single-cell genome sequencing data based on the k-mer approach**. The major steps are k-mer counting, normalization, computation of distance matrices and phylogeny building.

We describe our strategy through a series of steps performed in order. An overview of our method is presented in Figure 1. For our purposes, a "sample" is a single tumor cell genome and we use both terms interchangeably in the text.

### Starting data

We assume the input genome sequence data consists of quality-controlled short sequencing reads from which sequencing artifacts including adapters, polymerase chain reaction (PCR) primers or chimeras have been removed.

### Step 1: Deriving the k-mer count matrix

Our reference-free approach relies on using genome sequence information encoded in discrete genome marker units called k-mers, or sequences of DNA of length k. For small values of k, we anticipate finding all possible k-tuples of the DNA alphabet {A,T,G,C} in a sample. As k increases, we expect not to fully explore the k-mer space as the size of the human genome would limit the occurrence of all combinations of letters in the alphabet. For example, DNA sequences of length 18-24 have been shown to have unique specificity in the genome [23,24]. If the genome were completely unique, we would expect to see N - k + 1 unique k-mers where N is the average genome size of 3 × 10^9^ base-pairs. We use a k-mer counter to generate counts of occurrences of each unique k-mer. A tumor genome can then be represented by a vector of k-mer counts. We aggregate count vectors from all samples into a k-mer count matrix which can be processed further for downstream analyses.

### Step 2: Noise correction and normalization

There are several contributors to noise in deep sequencing data that must be considered in the model.

1 Noise due to sequencing error: Each sequencing technology has an inherent error rate for base-calling and this is usually an error probability or score for each base of the read. For example, Illumina Fastq files come with phred [25] scores for each base. A major consequence of such sequencing errors is confounding when calling sequence variants since distinguishing true variants from sequencing errors is a challenge. We assume that such sequencing errors would occur in a much smaller fraction and correct for it by filtering out k-mers with sparse occurrences.

2 Noise due to technical variation: It is widely known that sequencing depth may vary by sample or subsets of samples. The number of reads for each sample may be different due to varying experimental conditions, true differences in abundance of DNA copies, nature of the contig, repeats or microsatellite regions, or other sources of noise. Typically, to allow for even comparison in the absence of technical variation, counts are either normalized [26] or adjusted for during analyses (e.g., sequencing depth is included as a covariate during multiple regression). We normalize the counts using Total Sum Scaling (TSS) where the count of each k-mer is divided by the total sum of all k-mers considered in that sample to derive a compositional matrix of k-mer relative abundances for each sample. A global normalization method like quantile normalization applied to the counts directly would be less favorable in tumor data as individual samples can have widely varying genomic content. In the absence of ploidy information, we may fail to effectively differentiate between changes in genome ploidy and other forms of local copy number changes.

3 Noise due to random amplifications in the Whole Genome Amplification or WGA technology: Noise results from unevenness inherent in the amplification technique and as a result, certain regions of the genome may be amplified in some samples and not in others. Since WGA was performed up-stream of sequencing to generate our test dataset, we address this unique technical limitation by only including those k-mers that are amplified or "occur" in all samples. By imposing this strict condition, we limit ourselves to a much smaller section of the genome that will be available for analyses and, thus, preferentially select for amplifications and genome ploidy multiplications at the cost of detecting truly deleted or missing genomic regions. We also cannot identify true structural rearrangements occurring in a subset of samples. For sequencing technologies not using WGA, we recommend a less stringent filtering method based on occurrence of k-mers in a certain fraction of samples or by a measure of variance of individual k-mer count measures.

4 Noise due to alignment: The massive structural rearrangements accompanying tumor progression makes alignment to reference genomes noisy and non-robust. As our method is reference-free, we do not deal with this source of error.

### Step 3: Dimensionality reduction and feature selection

When k increases, the computational expense of analyzing an exponentially increasing k-mer count matrix overpowers the computational efficiency of k-mer counting. In such a scenario, dimensionality reduction by selecting informative k-mers both improves downstream analyses as well as computational complexity. Depending on the downstream application, we explore a few different strategies for dimensionality reduction. Because our data was derived from WGA assays, we only select k-mers present in all samples. In the absence of this technical limitation, we propose an alternate procedure of unsupervised filtering [27] based on minimum variance or interquartile range (IQR) on normalized data (counts or compositional). We further select informative k-mer features by testing for differential abundance followed by adjustment for the multiple testing. Such a reduced k-mer feature set can be further probed for classification or functional relevance.

### Step 4: Applications: classification and phylogeny inference

We present two applications for k-mer feature sets derived from k-mer count matrices in this paper: (1) predictive classification of tumor cells into different stages of progression and (2) inference of tumor phylogenies. Assuming we have samples with different class labels - different stages of tumor progression or case-control designs, the reduced set of informative features can be used to build classifiers for predictive classification of cells.

The second application is in tumor phylogeny inference. Interpretation of phylogenies depends on the characteristics of the starting data. Character-based phylogenies use discrete representations of features for each sample going into the phylogeny, usually a vector of binary features. Selecting robust markers of progression and discretizing them are usually challenging steps. The resulting phylogenies are easier to interpret as each split in the tree represents changes in specific features that underly the transitions between nodes, e.g., coordinates of copy number change, single nucleotide variations, etc. Distance-based phylogenies are simpler in that they start with a matrix of similarities between individual samples but the non-leaf nodes are harder to interpret in terms of specific changes in features that lead to inter-node transitions. Distance-based trees thus provide a global picture of evolution. Here, we restrict ourselves to distance-based phylogenetics and derive evolutionary trees using distance matrices computed from the k-mer count matrix.

### Demonstration on real single nucleus sequencing data

To illustrate our method, we downloaded the raw sequence reads from genomes of 200 breast tumor cells [18] from the NCBI sequence read archive (SRA) [28] as fastq files. 100 cells came from a primary ductal breast carcinoma patient; referred to as T10. The remaining 100 were a combination of 48 liver metastatis cells and 52 primary breast tumor cells, referred to as T16M and T16P respectively, from a second patient T16. Matched normal cell data was not available. The original single nucleus sequencing experiment employed whole genome amplification (WGA) with random priming resulting in deep sequence reads with coverage of 138 reads per bin of reference genome mapped [18] and an average read length of 48 bp. Amplification artifacts were ignored for lack of information on PCR sequences. We checked sequencing base quality control using FastQC [29] and found varying quality. Instead of trimming poor quality reads, which is often used as a quality control step, we correct for this error source by filtering out sequences with low abundances, as we would expect with reads containing sequencing errors. Due to the short size of reads, FastQC could not identify any Illumina single-read adaptors that we provided in the configuration file. We expect to lose k-mers uniquely coding for adaptors during the k-mer selection and filtering stage.

### k-mer inference

We used the fast k-mer counter Jellyfish [30] to count k-mers of lengths 5, 10, 15, 20 and 25 from the individual cells. The command *jellyfish count -c k -o output -t 32 input.fastq *creates the k-mer count hash, and the command *jellyfish dump -c output *recovers the count of each k-mer from the hash. The hashes from all cells were then merged to generate the count data matrix. The rows of the matrix are k-mer features and the columns are individual samples. The counts were normalized using TSS as described earlier to derive count fractions. Only k-mers present in all samples were retained for downstream analyses.

### Data survey

For an exploratory analyses of data substructure in the k-mer space, we perform ordination by the method of t-Distributed Stochastic Neighbor Embedding (t-SNE) [31] which is capable of capturing manifolds and non-linear substructures. We first generate dissimilarity matrices from the compositional matrix of k-mer relative abundances using the Bray-Curtis dissimilarity measure. Bray Curtis dissimilarity is commonly used for handling compositional data matrices in molecular ecology [32] and is hence, an appropriate measure in our context of k-mer relative abundances. We use the R package *vegan *for the dissimilarity computation and *Rtsne *for ordination. We additionally visualize the k-mer occurrence space by means of histograms.

### k-mer feature selection

We used minimum interquartile range (IQR) filtering and filtered out k-mers with IQRs in the first quartile of all k-mer IQR values. We chose the non-parametric Wilcoxon's rank-sum test to identify filtered k-mers differentially abundant in primary and metastatic stages of tumor progression. To adjust for multiple hypothesis testing, we used a conservative Bonferroni correction on a p-value cutoff of 0.1%. This step of global feature selection is important both for dimensionality reduction as well as for deriving a small subset of informative k-mer features. Such a set can be further validated by meta-analyses for consistent differential abundance in addition to use in various downstream applications. We describe methods for two such applications in next two subsections. All statistical analyses was done using the R language for statistical computing (version 3.1. or higher).

### k-mer counts as features for learning tasks

To test the discriminative power of the selected k-mer features to classify the tumor cells as primary or metastatic, we performed classification tasks on the k-mer feature count distribution matrix. We used two classifiers -- support vector machines (SVMs) and classification decision trees -- for comparison using 10-fold cross validation to avoid overfit. We used the SVM function in the *e1071* R library and the rpart function in the *rpart *library for model-fitting and class prediction. We assessed performance by computing average classification error for 10 replicates of 10-fold cross-validation.

### Distance-based phylogeny reconstruction

We computed Euclidean distance matrices in which each non-diagonal matrix element is a measure of evolutionary distance between two samples. Thus, when comparing across samples, we are comparing fractions of the genome occupied by different k-mers which approximately captures the differences in genome composition across the samples. Neighbor-joining trees were built using *neighbor *program in PHYLIP[33]. 50,000 bootstrap replicates were used to construct consensus neighbor joining trees.

### Analyses of resulting phylogenies

In the absence of ground truth for comparisons, we defined a test statistic for analyzing the phylogenies that would capture how well the tree partitions cells belonging to different stages of tumor progression. We would expect cells belonging to the same stage from the same tumor to be clustered closer together than cells from different tumors or stages. We defined a test statistic that would serve as the metric of separation, to be the ratio of the average distance between cells in the same class and the average distance between cells in different classes. We then sought to reject the null hypothesis that cells are randomly distributed in the phylogeny. We performed 10,000 permutation tests to derive the distribution of the test statistic for the null hypothesis. We ascertain p-values at a significance threshold of 0.001 for interpretation.

$$\text{Test}\;\;\text{Statistic}=\frac{\sum \text{pairwise}\;\;\text{distances}\;\;\text{between}\;\;\text{cells}\;\;\text{in}\;\;\text{the}\;\;\text{same}\;\;\text{class}}{\sum \text{pairwise}\;\;\text{distances}\;\;\text{between}\;\;\text{cells}\;\;\text{in}\;\;\text{different}\;\;\text{classes}}$$

## Results and discussion

### Data survey

**Figure 2:**
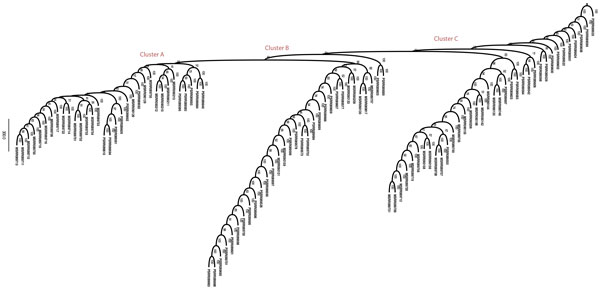
**Histogram of k-mer relative abundances**. Both 20- and 25-mer relative abundance densities appear log-laplacian. These data included 20- and 25-mers found in all tumor cells. (a) Histogram of 20-mer relative abundances in log10 scale. (b) Histogram of 25-mer relative abundances in log10 scale.

We demonstrate our methods through the analyses of the breast tumor single nucleus sequencing data [18] described earlier. We used Jellyfish to count k-mers. As k increases, the size of the hashes per sample also scale non-linearly. Combining hashes of all cells further increases data matrix file sizes. For example, when k = 25, the merged table is as large as 3.6TB. Since the k-mer count matrices tend to get sparse with increasing k, data subsampling can effectively reduce the matrices to sizes that can be easily manipulated. As described in the preceding section, we reduce the size of the matrices by only keeping those k-mers present in all samples. Table 1 describes the distribution of k-mer counts with expected and observed occurrences of unique k-mers. As k increases, the number of unique k-mers actually found in the samples decreases as we would expect the size of the genome to be a limiting factor. While the true number of k-mers would be expected to saturate around the length of the non-repetitive regions of the genome, a smaller fraction of these will be observed as the k-mer length approaches the length of the sequence read. Our strict criterion of screening out k-mers not found in all samples would be expected to greatly amplify the loss of k-mers as they get longer, either because of chance mutations in one or more samples, failure to amplify the entire region in one or more samples, or failure to produce a read covering an entire k-mer. On normalization by Total-Sum-Scaling, the distribution of compositional values appears log-Laplacian, as seen in Figure 2. During quality control, we noticed two metastatic cells from T16 have identical k-mer counts across values of k and, hence, removed one sample from downstream analyses.

**Table 1:** Distribution of k-mer counts found in the breast tumor single-nucleus sequencing data.

k-mer	Size of k-mer space	Observed k-mer space	Reduced space of k-mers
5-mer	4^5 ^= 1024	1024	1024

10-mer	4^10 ^= 1048576	1048576	1002150

15-mer	4^15 ^= 1073741824	1010206155	749386

20-mer	4^20 ^= 1.0995 × 10^12^	5667547542	128425

25-mer	4^25 ^= 1.1259 × 10^15^	1087589480	11966

Column 1 lists the different "k"s explored. Columns 2 and 3 describe the total number of possible unique k-mers for a given k and the actual number of unique k-mers observed in the data respectively. Column 4 describes the k-mer counts after adjusting for sparsity and amplification noise.

**Figure 3:**
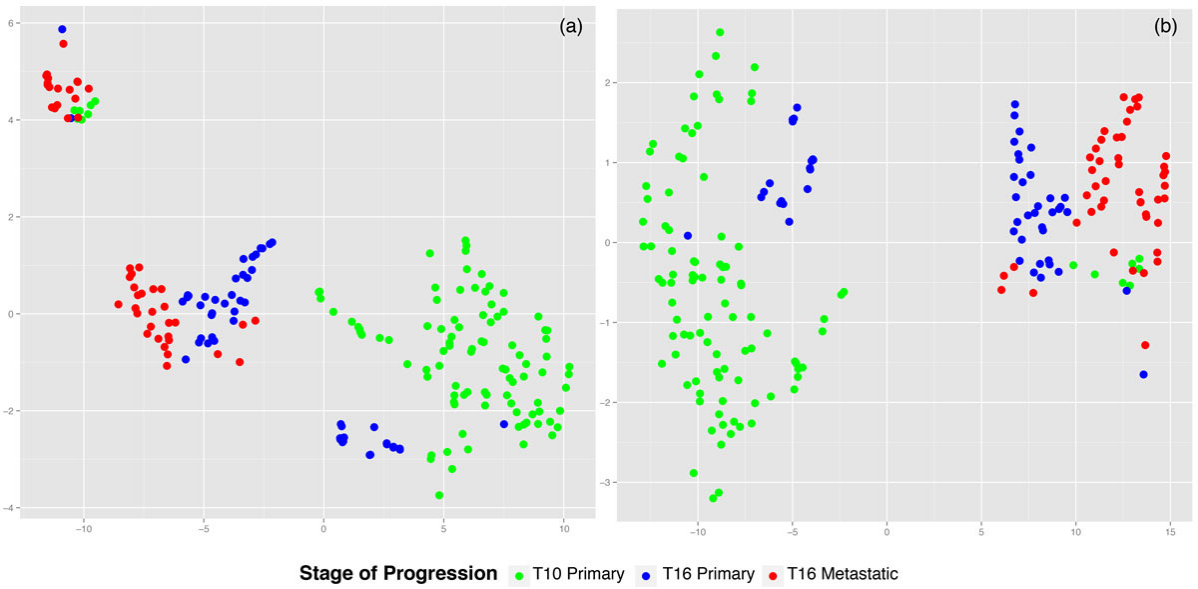
**tSNE ordination of single tumor cells**. (a) Projection of tumor cells in the space of 20-mers present in all cells. The 20-mers separate the cells into 3 loose groups, the rightmost of which is dominated by primary cells and the others by metastatic cells. The cluster in the middle is equally represented by primary and metastatic cells from patient T16, suggesting a state of transition. The cluster on the upper right is mostly metastatic with some primary cells. This suggests two distinct stages of advancing tumor cells based on k-mer composition. (b) Projection of cells in the space of only those 20-mers that remain after the differential abundance test for k-mer selection. Cells group into 2 clusters, one of which is entirely composed of primary cells and the other a mix of primary and metastatic cells.

Figure 3(a) shows ordination of the tumor cells based on 20-mer relative abundances in 2-dimensional space. The tumor cells spread into three distinct clusters. There is one cluster comprised mostly of primary breast tumor cells from patient T10 along with representative primary cells from patient T16. A second cluster is comprised entirely of cells from patient T16 with a mix of primary and metastatic stages of progression. The third cluster is most interesting, as it is dominated by metastatic cells from T16 but has a fair share of primary cells from T10 and a few T16 primary cells suggestive of a stage in transition from primary to metastatic across patient samples. The ordination suggests that the k-mer count distributions may by themselves have information to effectively separate the data into meaningful phenotypes.

### k-mer feature selection

Using the minimum IQR filtering approach described above, we found a 25% reduction in both 20-and 25-mer space respectively resulting in 96319 20-mers and 8795 25-mers respectively. Differential abundance testing using a Wilcoxon rank-sum test further reduced the space by 48.2% and 38% for 20-and 25-mers, respectively, at a Bonferroni-corrected p-value significance threshold of 0.001. This results in a selected k-mer feature set of 49874 20-mers and 5549 25-mers. Figure 3(b) shows the ordination of samples in the reduced k-mer feature space. The samples appear separated into two groups, one groups composed entirely of primary cells, and the other has all the metastatic cells from T16 and a smaller set of primary cells from T10 and T16, potentially in more advanced stages of primary tumor evolution.

We used NCBI BLAST [34] to query a few 25-mer sequences against the database of sequences of known cancer genes in order to determine specific hits of functional significance. ERBB2 is a gene which is commonly over expressed or amplified in breast tumori-genesis is a well known breast tumor marker [35,36]. We report 3 25-mers which align to the ERBB2 gene with a single mismatch in Table 2. While there may be interesting information to mine in the remaining 25-mers, we tested only for the presence of a known amplicon we expected to find for validation purposes. We would expect that most biases in k-mer distributions would simply reflect random CNV diversification, but there may be other amplicons or regions of loss of heterozygosity that may be discovered in a detailed functional analysis.

**Table 2:** Ascertaining possible functional roles for k-mers.

Examples of 25-mers that align to the ERBB2 gene	Location on ERBB2 gene (bp)
TCACCCAGGTTGGAGTGCAGTGGCA	9592-9616

CACACCTGTAACCCCAGCACTTTGG	16172-16196

CACTCTAGCCTGGGCGACAGAGCGA	36185-36161

Column 1 lists 3 25-mers that aligned to the ERBB2 gene with a single-base difference in DNA sequence. ERBB2 is one of the most frequently amplified genes in primary breast cancer and a widely recognized tumor progression marker.

### Classification performance

10-fold cross-validation with 10 replicates gave us median prediction errors of 0.0053 for both SVMs with linear basis functions and classification decision trees (CART). Both methods classify the cells with very high accuracy. Given the uneven class distribution of cells (ratio of primary to metastatic cells is 3:1), we expect the overall accuracy to be slightly exaggerated. So, we subsampled the primary cells to yield even class distribution and performed the classification tasks described above for 10 replicates. The median prediction errors for SVMs and CART continued to remain high at 0.02 and 0.016. We report the classification performance error in Table 3. The relatively high accuracy is suggestive of the utility of k-mer based features for staging and classification.

**Table 3:** Performance of SVM and classification trees.

Classifier	Class Distribution	Min	Median	Mean	Max	Standard deviation
SVM	Uneven, all samples Even, subsampled	0.003 0.003	0.005 0.02	0.006 0.02	0.01 0.03	0.002 0.008

CART	Uneven, all samples Even, subsampled	0.004 0	0.005 0.016	0.008 0.02	0.016 0.04	0.002 0.013

Values shown are classification errors computed as the average ratio of incorrectly classified test samples to the total number of test samples per replicate. We report results both including the entire dataset and results from 10 replicates of subsampling to ensure an even class distribution.

### Distance-based phylogenies

**Figure 4:**
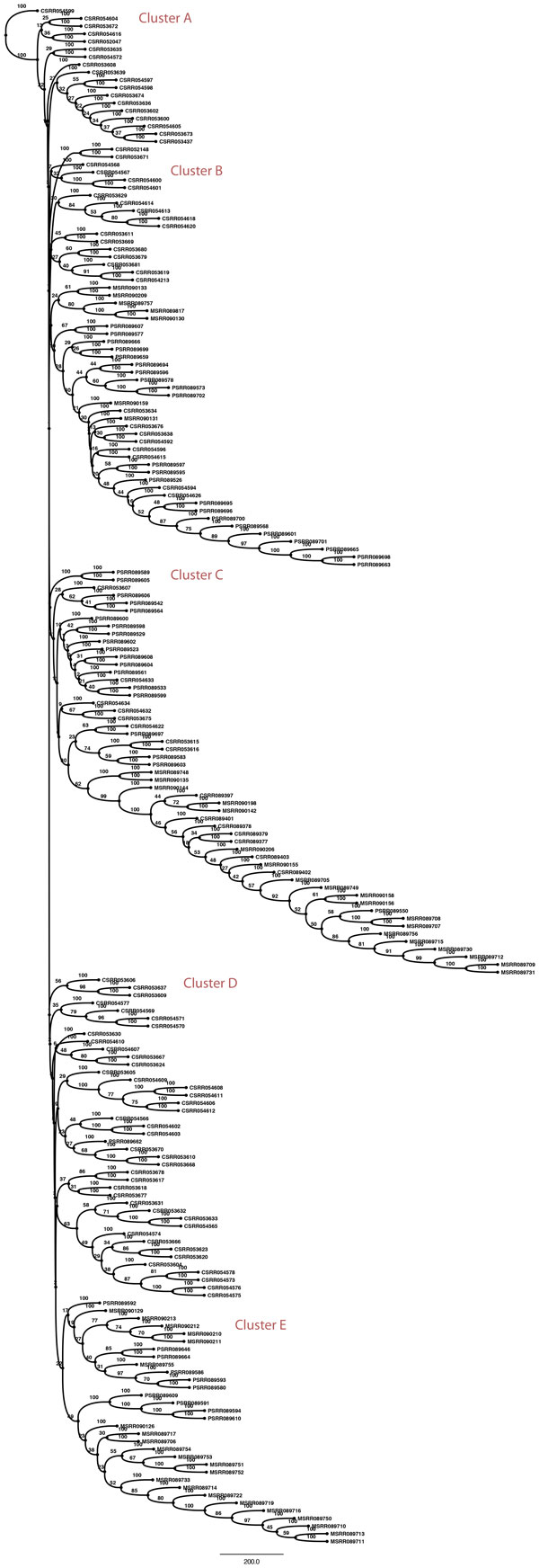
**20-mer bootstrap consensus neighbor-joining tree built from T16 primary (prefix P) and metastatic data (prefix M)**. Distinct groupings of cells are labeled as clusters.

**Figure 5:**
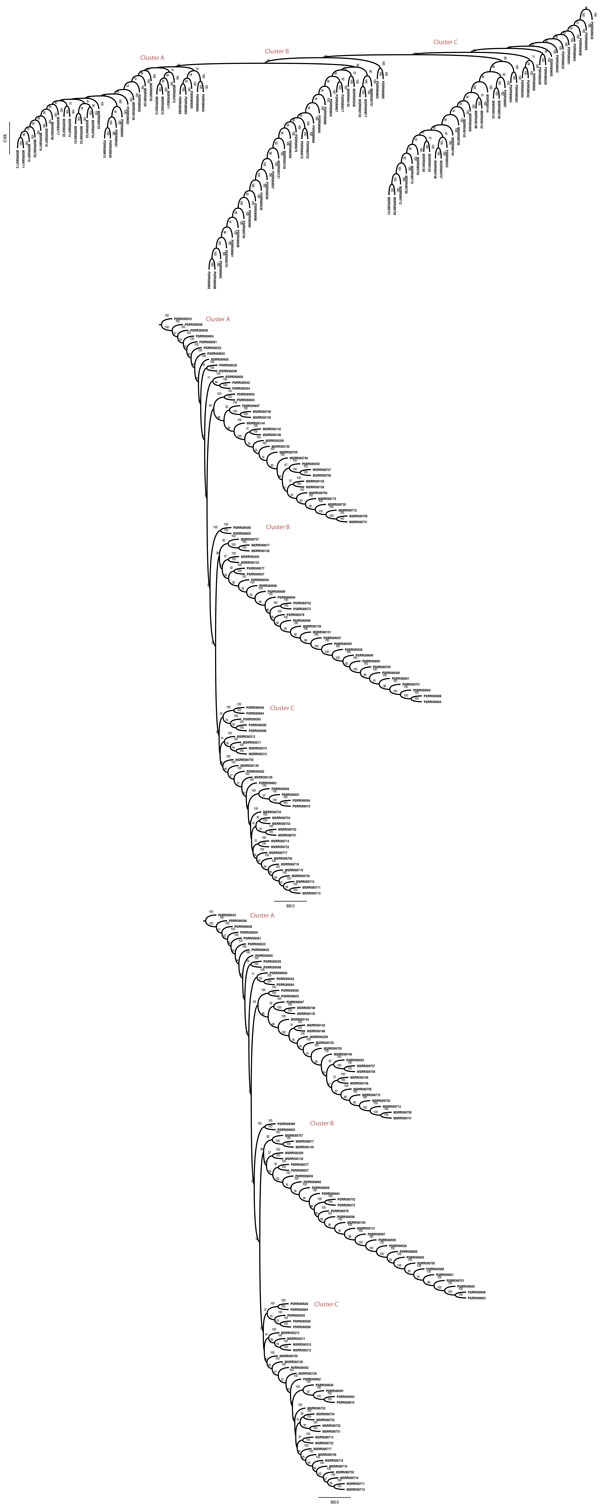
**20-mer bootstrap consensus neighbor-joining tree built from T10 primary breast tumor cells (prefix C), T16 primary (prefix P) and metastatic data (prefix M)**. Distinct groupings of cells are labeled as clusters.

We report phylogenies for k = 20, which yielded the largest number of distinct kmers after filtering. Figure 4 shows the bootstrap consensus tree constructed using all cells from both samples. We may distinguish among 3 classes: primary cells of T10 (prefixed C), primary cells of T16 (prefixed P), metastatic cells in T16 (prefixed M). There are 5 broad clusters of cells based on phenotype: Clusters A and D are entirely composed of primary cells from T10. Cluster E is similarly entirely composed of cells from T16. Cluster C is mostly a primary cluster dominated by T10 primary cells and a few metastatic cells, potentially early in their infiltrating stage. Cluster B is a primary cluster with cells from both patients and a small number of metastatic cells. The phylogenies in the original tree [18] have 3 distinct clusters for T10 based on ploidy and we observe that T10 cells are found in 4 distinct clusters, two of which are entirely filled with T10 cells. While it is hard to determine the biological meaning of the placement of tumor cells because of the lack of ground truth, we test for the quality of clustering by statistical analysis using the test statistic described in Methods. The test statistics obtained for k = 5, 10, 15 and 20-mer trees are described in Table 4. In all cases, the values had a p-value of less than 0.0001 when compared to the mean value obtained from the permutation tests. This confirms that the placement of samples is the tree is non-random. The phylogenies in the paper that originally generated the data used [18] were built by aligning the genomes to a reference. A full head-to-head comparison to their approach is not feasible because (1) information on leaf node labels are not available (2) the authors color leaf nodes by ploidy and this information cannot be matched to the cell names provided in SRA and (3) we do not actually know the ground truth answers. While lack of a ground truth is often handled in the phylogenetics literature through simulated data, technologies for single-cell sequencing are sufficiently new and advancing sufficiently rapidly that we do not have simulation models one can trust to faithfully capture the challenges of real data. To better address these issues despite the challenges, we have conducted an indirect comparison using phylogenies built per patient and looking at the number of major clusters and ordering of cells on the tree for T16 to check for similarities to the clusterings found in the original paper. Figure 5 shows the bootstrapped consensus neighbor-joining tree constructed from both primary and metastatic cells in T16 for k = 20. We observe 3 distinct clusters: Cluster A has sub-clusters, majority of which have no mixing and are either entirely metastatic or primary. Cluster B is dominated by primary cells. Cluster C is a more even mix of primary and metastatic cells. The original paper has neighbor-joining trees built based on copy number profiles and breakpoint regions for the two patients individually. The trees in the original paper for T16 have two major clusters colored by ploidy, both of which are a mix of primary and metsatatic more similar to Clusters A and C. Our phylogenies show a more granular clustering scheme. While we cannot definitively say our results are better or worse than theirs, our analyses do show that they yield comparable groupings of cells. We can therefore establish that our method yields a qualitatively similar partitioning of cells to Navin et al.'s [18] reference-based method, while avoiding the computational issues raised by mapping to a reference genome.

**Table 4:** Results of permutation tests to assess phylogenies.

k-mer	Test Statistic	Distribution mean, sv	p-value
5-mer	0.6484	1, 0.0048	≤0.0001

10-mer	0.7333	1, 0.0058	≤0.0001

15-mer	0.6196	0.99, 0.0058	≤0.0001

20-mer	0.8266	1, 0.0049	≤0.0001

Column 2 is the test statistic that served to capture how well cells from the same stage of progression cluster. Column 3 describes null distribution characteristics derived from permutation testing. Column 4 is the probability of occurrence of the test statistic in the null.

## Conclusions

We have described a strategy to derive distance-based tumor phylogenies from whole genome sequencing data. k-mer counting provides an efficient way to go from genome sequences to informative markers of differences in genome copy numbers. In addition, selecting k-mer counts as features to represent genomic imbalances adds utility for practical applications of classification and staging. We show how evolutionary models derived from the k-mer relative abundances can be used to build phylogenies and demonstrate a method to analyze the resulting phylogenies as a measure of how well they partition different stages of tumor progression. While the current contributions are mainly methodological, future directions include applying the strategy to larger real datasets towards inferring biologically significant observations from the resulting phylogenies. The availability of single-cell sequencing data is still limited and the method is likely to have more potential, especially with regard to novel biological discovery, when more data with clinical covariates become available. Further, the list of k-mer features can be used in other applications not explored in the paper. For e.g., they can be further tested for consistent differential abundance through meta-analysis or specific experiments to gauge functional relevance.

We have demonstrated that genome dosage differences represented by k-mers can separate tumor progression phenotypes with reliable classification performance. Our results suggest that there is enormous redundancy in phylogenetically informative copy number variation across the genome and, in particular, that one does not need to see more than a small fraction of the genome in a solid tumor to identify cells from a given lineage. Extensive diversification by copy number variations is a common feature of solid tumors [37,38], so it is unsurprising that examining copy number status of a random subset of the genome would often allow one to robustly distinguish between two possible tumors of origin for a given sample. This observation has important implications for efforts at tumor phylogeny construction, since it argues that one can perform tumor phylogenetics reliably with fairly little data on individual tumor cells. Our k-mer approach is so far unusual for the field, though, in exploiting this enormous redundancy of phylogenetically informative variation in single tumors.

## Competing interests

The authors declare that they have no competing interests.

## Authors' contributions

Both A.S and R.S conceived the research idea and wrote the manuscript.

A.S implemented the workflow and performed experiments.

## Declarations

The publication of this article was funded by U.S. NIH grant R01CA140214 and Carnegie Mellon University.

This article has been published as part of *BMC Genomics *Volume 16 Supplement 11, 2015: Selected articles from the Fourth IEEE International Conference on Computational Advances in Bio and medical Sciences (ICCABS 2014): Genomics. The full contents of the supplement are available online at http://www.biomedcentral.com/bmcgenomics/supplements/16/S11.
